# Editorial of Special Issue “Pharmacomicrobiomics in Non-Communicable Disease”

**DOI:** 10.3390/biomedicines10071605

**Published:** 2022-07-06

**Authors:** Amedeo Amedei

**Affiliations:** 1 Department of Clinical and Experimental Medicine, University of Florence, 50139 Florence, Italy; amedeo.amedei@unifi.it; 2 SOD of Interdisciplinary Internal Medicine, Azienda Ospedaliera Universitaria Careggi (AOUC), 50139 Florence, Italy

The human superorganism, also known as the human holobiont, is a complex organism made up of host body as well as the bacteria, archaea, viruses, and fungi that live inside it along with their genes.

Since most terrestrial species exist as composites, humans are not exceptional in having a genetic structure that is primarily microbial. That is a fundamental characteristic of a large portion of life on Earth, which is a planet dominated by microbes [1].

The various microbiomes that live inside of our bodies in a variety of places (including the gut, skin, airways, urogenital tract, and breast tissue) provide vital metabolic, physiologic, regulatory, host defence, and colonization resistance functions, as well as vitamins essential to life.

The microbiome could be considered the first medication, indicating a 21st-century healthcare paradigm that emphasizes the entire host, the human superorganism, starting with the microbiome [2], as clearly described by Dietert R.R in this Special Issue.

Up until now, a large portion of medicine has safeguarded and cared for patients as though they were one species. As a result, microbiota have been harmed unintentionally, and chronic illnesses, including non-communicable diseases and conditions (NCDs), are on the rise. Loss of colonization resistance, greater vulnerability to infectious illnesses, and an increase in multimorbidity and polypharmacy over the course of life were all NCDs consequences. The human microbiota must take center stage if we are to advance toward sustainable healthcare.

Dietert described the relationship between the human microbiota and physiology from the perspective of systems biology governance, as well as the ongoing NCD epidemic, including the impacts of current treatments (as well as other variables) that harm the microbiota. Examples are shown for two NCDs with entrance points, obesity and asthma, and their vast networks of comorbid NCDs. Changes in even a few medical decisions that prioritize the microbiota could have a major impact on health across the life course under the concepts of microbiome-first medicine, considering the significance of keystone bacteria and crucial windows of development.

The study of how the microbiota and xenobiotic response interact, or how microbiota changes affect the efficacy and toxicity of treatments, is known as pharmacomicrobiomics. According to recent research, one in four of the meds that we regularly take to treat conditions other than intestinal diseases alter the gut microbiota (GM), leading to negative side effects and an increase in bacterial resistance. Additionally, it is well established that drug–microbial interactions primarily occur in the colon, where drugs may regulate microbial metabolism, alter the intestinal milieu, and affect bacterial development by changing the GM structure. Because of this intricacy, pharmacological research must use a molecular biology approach to understanding cell signaling and the intricate interactions between hosts, microbes, and drugs.

In order to comprehend how the microbiota is involved in drugs’ responses, it is crucial to look into the concept of pharmacomicrobiomics. This radical concept may open the door to a novel strategy in which the intestinal microbiota is the target to be modified in order to improve the therapeutic efficacy lessening the harmful consequences. In order to better understand the molecular mechanisms and how this new branch could result in a new treatment approach, the goal of this Special Issue is to present an overview of the interaction between pharmacomicrobiomics and NCDs.

Probiotics administration, which frequently demonstrated effective effects in patients and animal models of irritable bowel syndrome (IBS), is one of the well-known and out-of-date approaches to shape the microbiota [3]. Wang X et al. looked at how Bifidobacterium bifidum G9-1 (BBG9-1) affected the behavior of macrophages and colonic mucosa in rats that had their mothers separated (MS).

In comparison with controls, MS rats had considerably increased colonic permeability and less claudin 4 expression in the colonic epithelium. In addition, in the colonic mucosa of MS rats, the expression of IL-6 and IFN-γ increased along with a large increase in CD80^+^ macrophages. Finally, BBG9-1 therapy reduced the rise in M1 macrophage and IL-6/IFN- γ expression in the colonic tissue of MS rats, and simultaneously improved the enhanced mucosal permeability and the decreased claudin 4 expression.

Furthermore, several in vivo and in vitro studies have shown that Bifidobacteria and Lactobacilli, together with their cellular constituents, have great antioxidant potential, which offers a certain amount of protection to the human body against diseases linked to oxidative stress. Bifidobacteria and Lactobacilli are now being looked at as potential new sources of natural antioxidants. The article by Averina O.V et al. [4] highlighted the most recent findings in regards to the diverse antioxidant capabilities of Lactobacilli and Bifidobacteria.

The mechanisms of these bacteria’s antioxidant action in the human GM, which involve bacterial cell components and metabolites, are especially emphasised. The authors also stressed the genes responsible for the antioxidant capacities of specific strains of Lactobacilli and Bifidobacteria in order to assess their antioxidant potential in the human gut microbiota. In order to develop diagnostic tools for evaluating oxidative stress and to make decisions on how best to restore normal GM functioning and, in turn, human health, it is crucial to identify the antioxidant GM biomarkers. Finally, the authors covered the practical use of probiotic strains with demonstrated antioxidant capabilities to prevent oxidative stress.

The effects and potential action mechanisms of probiotics, prebiotics, and symbiotics in dermatological disorders such as atopic dermatitis (AD), psoriasis, chronic ulcers, seborrheic dermatitis, burns, and acne were instead examined by Karina Polak et al. [5]. The authors opted to include case reports and original studies about oral administration and topical application of the pro-, pre-, and symbiotic in the final analysis due to the extremely small number of studies available on the topic of GM modulation in all skin diseases other than AD. The majority of the examined studies either showed promise in animal or ex vivo models or significantly improved patient health. The topic of microbiota shaping as a treatment strategy for skin illnesses nevertheless calls for more research due to the paucity of available data and clear findings.

The onset of numerous human pathologies associated with an unbalanced inflammatory process, including cardiovascular diseases like myopericarditis, which were the subject of the review by Andrea Piccioni et al. [6], is linked to taxonomic and/or functional dysbiosis, as has been previously reported and well documented.

Myopericarditis is primarily caused by viral infections, while it can also result from bacterial infections, toxic chemicals, and immune system problems. All of these illnesses can cause serious inflammation and myocardial damage, which are frequently accompanied by a negative prognosis. It is still unclear and under investigation what exactly these various pathogens—in particular viruses—do, how they interact with the host, how they affect the gut microbiota, and how the immune system reacts to them. It is interesting to note that several studies have shown the role of the gut microbiota and its related metabolites—some of which can mimic the cardiac myosin—in the myopericarditis development and cardiac inflammation.

They may cause an ongoing, ineffective immunological response that damages the myocardium through inflammation. Based on the hypothesis that GM modulation can be a new frontier in the cardiology area to prevent or treat inflammatory cardiomyopathies, the authors thoroughly explored the GM involvement in myopericarditis, particularly for the cardiovascular implications of COVID-19 viral infection.

In this scenario, Curini L. et al.’s study addressed the heart disorders highlighting how the microbiome actually contributes significantly to their development [7]. In order to increase the treatments’ effectiveness by directing their actions, the authors also looked into pharmacomicrobiomics and the GM role in this process. They paid special attention to cardiovascular disorders and novel prospective treatments.

Instead, the work by Rahim M.I. et al. that looked at the effects of chronic peri-implant inflammation on the systemic expression of inflammatory cytokines [8] is intriguing in terms of the mechanisms contributing to inflammation. They revealed that in a mouse model, interferon expression was species-specific, lasted longer with implants present, and significantly decreased upon dual species infections following infection of subcutaneous implants with Streptococcus oralis, Aggregatibacter actinomycetemcomitans, Porphyromonas gingivalis, and Treponema denticola (alone or in combination).

As a conclusion, Bilello J. et al. undertook a review of all published studies that we are aware of that address the impact of pharmacological drugs, environmental modifications, and surgical intervention on the microbiota of the upper gastrointestinal tract [9]. Different regularly used prescription and over-the-counter medications have different effects on the upper gastrointestinal system. In more detail, the proton pump inhibitors may have less of an impact on the proximal esophagus and may influence the relative prevalence of some species in the lower esophagus. Some esophageal disorders are correlated with changes in the esophageal microbiota. The microbiomes of the esophagus and stomach have been demonstrated to be impacted by weight-loss medications. Changes in the gastrointestinal tract’s microbial community are linked to common surgical procedures. Geographical differences are correlated with changes in the gastrointestinal tract’s microbiome, indicating that environmental influences have an impact on the microbiome in the upper gastrointestinal tract.

In other words, developing therapeutic strategies to lower disease morbidity will be made easier by studying the impact of environmental and pharmaceutical alterations on the microbiome of the upper gastrointestinal tract.
